# IUC23442-80 Role of renal nephrometry score in predicting peri-operative outcomes following partial nephrectomy

**DOI:** 10.1093/oncolo/oyaf248.044

**Published:** 2025-08-29

**Authors:** O V Potdar

**Affiliations:** Mumbai, India

## Abstract

**Background:**

Renal cell carcinoma is the 13th most common malignancy in the world.^1^ The prevalence of disease is more common in Western countries compared to Asian countries, with the prevalence of disease ranging between 1.1 and 6 per 100000 in the Asian population.^2^ RCC is more common in males than in females.^3^ To study the usefulness of RENAL nephrometry score in predicting peri-operative outcomes in patients with renal masses undergoing partial nephrectomy.

**Methods:**

A prospective single-center observational study was carried out at a tertiary care center in western suburbs of India for a period of 18 months, where all patients of renal masses satisfying inclusion and exclusion criteria undergoing Nephron sparing surgery were enrolled on the study and data were collected and analyzed.

**Results:**

In our study, renal masses with RENAL Nephrometry Score above 9 had a greater tendency for open surgical approach, greater warm ischemia time and total operative time, greater blood loss, and longer hospital stay with increased post-operative complications.

**Conclusions:**

Our study demonstrated that RENAL Nephrometry score is a useful tool in predicting surgical approach, warm ischemia time, total operative time, estimated blood loss, length of hospital stays, progression to total nephrectomy, and post-operative complications.

